# Transient Insanity

**Published:** 1849-04-01

**Authors:** 


### TRANSIENT INSANITY.

We copy the following observations from the *Spectator* of March 10th, a paper which has always taken a prominent and praiseworthy position among the newspaper press of this country in its advocacy of philosophical and psychological truths. The poor woman who was represented to have been, when she threw her son into the Regent's-canal, in a state of "wild excitement," undoubtedly was suffering from an attack of transient insanity, using the term insanity as indicative of that condition of mind so often associated with loss of all power of self-control. Cases of this nature are not of uncommon occurrence. Many a man commits suicide under the influence of an attack of insanity, of the existence of which his most intimate friends have not the slightest idea. Persons labouring under the effects of terrible delusions have been known to conceal the fact for months. Those most closely allied to them—on terms of intimate and close association—apparently possessing their confidence, have been kept in ignorance of the existence of anything resembling a morbid state of mind. We have been consulted by persons who have confessed to us that they have been struggling unknown to any one but themselves for months with a morbid desire to take away human life! Others under the influence of concealed false perceptions have manifested strong suicidal impulses. Many a man destroys himself whilst in a state of insanity, giving no indication of any morbid condition of mind. We have often been amazed at the degree of control which many lunatics are capable of exercising over their delusive impressions. We have no right invariably to conclude, in cases of self-destruction, that the mind is unclouded and free from insanity or delusion, because the party has given no clear proof, prior to death, of the presence of mental aberration. We have no doubt that many crimes —for which the severest penalty of the law has been inflicted—many suicides, have been committed under the overpowering influence of some terrible delusion known only to the parties themselves! The brain is subject to occasional attacks of temporary disturbance, during which the mind, for a short period, is thrown off its balance, the person so affected being insane. This is what we understand by the term *transient insanity*. A gentleman who had been exposed to great anxiety of mind, impairing his general health, became affected with melancholia. Apart from his depression of spirits, the party in question gave no evidence of insanity. This gentleman was sitting one day with his wife, when he suddenly jumped up from his chair, exclaiming, in wild excitement, "Fly, for your life! Fly!" His poor wife, without saying one word, instantly left the room. He felt a sudden and, to him, unaccountable impulse to beat his wife's brains out with a poker. In a few minutes the attack subsided, and he reflected with horror on the dreadful position in which he had been placed. Not many months back, a gentleman of fortune, happy in his domestic circle, apparently without a care or anxiety to annoy him or ruffle his temper, deliberately stood before the glass and cut his throat! Who would be so bold as to declare that this unfortunate man's mind was free from disease? We purpose, in an early number, to consider the important subject of transient insanity more in detail.

"A verdict at the Central Criminal Court exemplifies at once the great advance made in the right understanding of matters that come within the cognizance of the criminal law, and the very imperfect state of the law itself. Anne Mallandine was tried on the charge of attempting to murder her son. She was an unmarried woman, twenty-eight years of age; the child was a boy of six or seven. She was seen to throw him into the Regent's-canal, at Haggerstone; and she would have plunged in herself, but a passenger came up and prevented her. The boy was rescued, and she was detained. She then proved to be in a state of wild excitement, brought on by distress. Her counsel, Mr. Cooper, suggested to the jury, that the evidence disclosed such a state of mind as did not amount to actual insanity, but prevented her from being aware of the effect of what she was doing. On that argument, apparently, the jury pronounced a verdict of acquittal.

"The ground on which Mr. Cooper obtained this verdict has the merit of going to the kernel of the matter; and the decision of the jury is as near an approach to the true conclusion as our law will permit; but still it is a very imperfect approach. It comes nearer than a verdict of 'guilty,' with 'a recommendation of mercy,' because that hands the prisoner over to the discretion of the judge, who sometimes pays no attention to the qualification. And it is evident that the jury did not hold the woman to be so accountable for her act as to deserve punishment. Yet there are grave objections to a simple acquittal in the case of a person *not* insane, but proved to have attempted murder. It opens a wide and difficult question as to the line between criminal passion leading to a criminal act, and excitement originating in an innocent cause but resulting in a criminal act—between the brief madness of anger and the brief madness of despair. If the jury had adopted Mr. Cooper's view—and the evidence left no alternative except that view or a verdict of guilty—they had come to a conclusion which the state of the law did not empower them to record. Multiply such verdicts, and you open the door of release to a dangerous class of persons who are excitable to the perpetration of outrage: on the other hand, a wiser perception of truth and justice will more and more forbid juries from affirming guilt where there is no moral accountability, or accountability in a very imperfect degree. They ought to be enabled to make a true record of the right judgment, and to hand over the prisoner for a treatment suitable to the case."

---

